# *N* ^6^-Substituted AMPs Inhibit Mammalian Deoxynucleotide *N*-Hydrolase DNPH1

**DOI:** 10.1371/journal.pone.0080755

**Published:** 2013-11-19

**Authors:** Claire Amiable, Sylvie Pochet, André Padilla, Gilles Labesse, Pierre Alexandre Kaminski

**Affiliations:** 1 Institut Pasteur, Unité de Chimie et Biocatalyse, Paris, France; 2 CNRS, UMR3523, Paris, France; 3 Université Paris Descartes Sorbonne Paris Cité, Paris, France; 4 CNRS, UMR5048, Centre de Biochimie Structurale, Montpellier, France; 5 INSERM, U554, Montpellier, France; 6 Université Montpellier 1 et 2; IFR3, Montpellier, France; Rutgers University -New Jersey Medical School, United States of America

## Abstract

The gene *dnph1* (or *rcl*) encodes a hydrolase that cleaves the 2’-deoxyribonucleoside 5’-monophosphate (dNMP) *N-*glycosidic bond to yield a free nucleobase and 2-deoxyribose 5-phosphate. Recently, the crystal structure of rat DNPH1, a potential target for anti-cancer therapies, suggested that various analogs of AMP may inhibit this enzyme. From this result, we asked whether *N* ^6^-substituted AMPs, and among them, cytotoxic cytokinin riboside 5’-monophosphates, may inhibit DNPH1. Here, we characterized the structural and thermodynamic aspects of the interactions of these various analogs with DNPH1. Our results indicate that DNPH1 is inhibited by cytotoxic cytokinins at concentrations that inhibit cell growth.

## Introduction

Nucleoside analogs are widely used in chemotherapy to fight against cancer and viral infections. Most of the anticancer nucleoside analogs exert their effect after incorporation into DNA. They interfere with one or more enzymes that are critical for DNA synthesis or DNA repair and cause DNA damage then apoptosis induction [1]. For example, purine analogs such as Fludarabine, Cladribine and Clofarabine used in the treatment of leukemia, are efficiently incorporated into DNA to act as chain terminators, but the latter two are also potent inhibitors of ribonucleotide reductase [2]. 

In fact, multiple steps of the nucleotide biosynthesis may also be targeted upstream from DNA synthesis and repair and multi-target strategy may help in preventing drug resistance. 

The plant hormone cytokinins are adenine derivatives with an isoprenoid or an aromatic side chain substituted at the N6 position. For instance, *N* ^6^-isopentenyladenosine, *N* ^6^-furfuryladenosine (kinetin riboside) and *N* ^6^-benzyladenosine have cytotoxic effects for different human cell lines derived from solid tumours [3–10]. These compounds also show good efficiency *in vivo* using several animal and xenograft models of cancer [4,5,11,12]. However, their actual cellular targets are unknown. 

While cytokinin bases induce differentiation at relatively high concentrations, their ribosides cause rapid apoptosis of leukaemia cell lines at low micro-molar concentrations [13]. Intracellular conversion of ribosides to their respective 5’-monophosphates by adenosine kinase is known to be required for their action [14–16]. This mode of activation resembles that of the triciribine, another pro-apoptotic nucleoside that is currently in phase I trials [17]. While the PH domain of Akt has been proposed as a target for triciribine 5'-monophosphate, we have recently shown that this compound also inhibits DNPH1 [18]. DNPH1 is a potential target for anti-cancer therapies as it is involved in cellular proliferation [19,20] and is up-regulated in several types of cancer [21–23].

DNPH1 is a 2’-deoxynucleoside 5’-phosphate *N-*hydrolase. It hydrolyses dNMP to form a free nucleobase moiety and 2-deoxyribose 5-phosphate [24]. The nucleotide binding and catalytic sites were probed with various synthetic nucleotide analogs and mapped using directed mutagenesis [25]. 

Given its catalytic activity and ligand recognition properties, DNPH1 can be considered to be a catabolic enzyme potentially involved in the maintenance of the balanced pool of nucleotides or in a detoxification process of modified nucleotides. Indeed, the structure of rat DNPH1 in solution [26,27] showed a largely open active site in the presence or absence of GMP. More recently, high-resolution crystal structures revealed how DNPH1 can accommodate distinct nucleotides such as the *N* ^*6*^-cyclopentyl AMP and the triciribine 5’-monophosphate used in anti-cancer therapies. This suggested that phytohormonal cytokinins may also target DNPH1.

Here, we report that various *N* ^6^-substituted AMP including some cytokinin derivatives inhibit mammalian DNPH1s, specifically rat and human DNPH1s, with affinity in the micromolar range. The X-Ray structures of three of these derivatives in complex with a recombinant variant of rat DNPH1 reveal the mechanism of the ligand recognition and suggest several routes for further optimization.

## Materials and Methods

### Chemicals

2’-Deoxyguanosine 5’-monophosphate was purchased from Sigma-Aldrich. *N* ^6^-Isopentenyl adenosine 5’-monophosphate (**1**), *trans-*zeatin riboside 5’-monophosphate (**2**), *cis*-zeatin riboside 5’-monophosphate (**3**), dihydrozeatin riboside 5’-monophosphate (**4**), kinetin riboside 5’-monophosphate (**5**) and *N* ^6^-benzyladenosine 5’-monophosphate (**6**) were from OlChemIm Ltd (Czech Republic). *N* ^6^-cyclopentyladenosine 5’-monophosphate (**7**) was from BioLog (Germany).

### General synthetic methods

 Reagents and anhydrous solvents were obtained from commercial suppliers and used without further purification. Flash chromatography was performed using silica gel 60 (Merck). Preparative HPLC was carried out on a Perkin Elmer system (200 Pump) with a C18 reverse phase column (Kromasil, 5µm 100Å, 250x10 mm) using a flow rate of 5.5 mL/min and a linear gradient of acetonitrile in 20 mM triethylammonium acetate buffer (TEAA) at pH 7 over 20 min. ^1^H, ^13^C and ^31^P NMR spectra were recorded on a Bruker Avance 400 spectrometer, operating at 400.13 MHz, 100.62 MHz and 161.62 MHz, respectively. ^1^H-^13^C-HMBC, HSQC experiments were performed for complete assignment of all signals. NMR spectra were reported in ppm (*δ*) and were referenced to the solvent residual signal or external standard (orthophosphoric acid for ^31^P). Coupling constants (*J* values) are reported in hertz. High-resolution mass spectra were recorded on a Waters Q-TOF micro MS instrument using a mobile phase of acetonitrile/water with 0.1% formic acid. The purity of all tested compounds was ≥ 97% as determined by HPLC analyses using a system (Agilent 1100) equipped with a diode array detector and using a reverse phase column (C18 Kromasil, 5µm 100Å, 150×4.6 mm,) and a linear gradient of acetonitrile in 10 mM TEAA buffer over 20 min at a flow rate of 1 mL/min. Retention time (*R*_*t*_) and gradient elution are given. 

### Synthesis of N^6^-alkyladenosine 5’-monophosphates (8-14)

AMP derivatives were synthesized in two steps from 6-chloropurine riboside according to reported procedures [28,29] Thus, 6-chloropurine riboside (58 mg, 0.2 mmol) was heated with an excess of the appropriate amine (3 equiv) in the presence of triethylamine (3 equiv) or calcium carbonate (3 equiv) for amines bearing an acid group at 60°C in absolute ethanol (2 mL). After evaporation of the volatiles, the crude mixtures were purified by flash chromatography on silica gel (eluent: 5% MeOH in dichloromethane) to afford the corresponding *N* ^*6*^-alkyladenosines. The 5'-phosphorylation of *N* ^*6*^-alkyladenosines (0,15 mmol) with POCl_3_ (3 equiv) in PO(OEt)_3_ (0.5 mL) at 4°C overnight afforded after purification by HPLC the expected nucleotides as triethylammonium salt. AMP derivatives **8-14** were isolated as sodium salt by ion exchange with Dowex 50W resin (Na^+^ form) in high purity (up to 97%). 

#### *N* ^6^-Cyclohexyladenosine 5'-monophosphate (8)

White powder (42 mg); *R*_t_ = 9.42 min (10-50% of CH_3_CN in 10 mM TEAA buffer); ^1^H NMR (DMSO-*d*_6_) δ 0.89-0.91 (m, 1H, H-4a cHex), 1.28-1.37 (m, 4H, H-2a cHex, H-3a cHex, H-5a cHex, H-6a cHex), 1.60-1.63 (m, 1H, H-4b cHex), 1.73-1.75 (m, 2H, H-3b cHex, H-5b cHex), 1.88-1.91 (m, 2H, H-2b cHex, H-6b cHex), 3.84-3.88 (m, 3H, H-5', H-1 cHex), 4.03-4.06 (m, 1H, H-4'), 4.21-4.23 (m, 1H, H-3'), 4.63 (t, *J* = 5.4, 1H, H-2'), 5.92 (d, *J*_1_’_,2’_ = 5.9, 1H, H-1'), 7.46 (d, *J*_1 cHex,NH_ = 8.4, 1H, NH), 8.19 (bs, 1H, H-2), 8.41 (s, 1H, H-8); ^13^C NMR (DMSO-*d*_6_) δ 25.4 (2C, C-3 cHex, C-5 cHex), 32.9 (2C, C-2 cHex, C-4 cHex), 64.8 (d ^2^,*J*_CP_ = 5.1, C-5'), 66.9 (C-1 cHex), 71.5 (C-3'), 74.4 (C-2'), 84.5 (d ^3^,*J*_CP_ = 7.5, C-4'), 87.2 (C-1'), 119.8 (C-5), 139.3 (C-8), 145.3 (C-4), 152.8 (C-2), 155.4 (C-6); ^31^P NMR (DMSO-*d*_6_) δ -1.18; HRMS (ESI-TOF) *m/z* calcd for [C_16_H_24_N_5_O_7_P+H]^+^ 430.1492, found 430.1482.

#### *N* ^6^-Cyclobutyladenosine 5'-monophosphate (9)

White powder (6 mg); *R*_t_ = 6.02 min (10-50% of CH_3_CN in 10 mM TEAA buffer); ^1^H NMR (DMSO-*d*_6_) δ 1.63-1.70 (m, 2H, H-3 cBu), 2.12 (bs, 2H, H-2a cBu, H-4a cBu), 2.25 (bs, 2H, H-2b cBu, H-4b cBu), 3.85 (bs, 2H, H-5'), 4.03-4.05 (m, 1H, H-4'), 4.21-4.23 (m, 1H, H-3'), 4.64 (t, *J* = 5.8, 1H, H-2'), 4.70 (bs, 1H, H-1 cBu), 5.92 (d, *J*_1_'_,2'_ = 5.8, 1H, H-1'), 7.95 (bs, 1H, NH), 8.19 (s, 1H, H-2), 8.45 (s, 1H, H-8); ^13^C NMR (DMSO-*d*_6_) *d* 15.0 (C-3 cBu), 30.7 (2C, C-2 cBu, C-4 cBu), 52.7 (C-1 cBu), 64.7 (d ^2^,*J*_CP_ = 5.0, C-5'), 71.6 (C-3'), 74.4 (C-2'), 84.5 (d ^3^,*J*_CP_ = 8.8, C-4'), 87.1 (C-1'), 120.0 (C-5), 139.6 (C-8), 146.9 (C-4), 153.0 (C-2), 155.0 (C-6); ^31^P NMR (DMSO-*d*_6_) δ -1.14; HRMS (ESI-TOF) *m/z* calcd for [C_14_H_20_N_5_O_7_P+H]^+^ 402.1179, found 402.1177. 

#### *N* ^6^-Cyclopropyladenosine 5'-monophosphate (10)

White powder (26 mg); *R*_t_ = 11.50 min (0-20% of CH_3_CN in 10 mM TEAA buffer); ^1^H NMR (DMSO-*d*_6_) δ 0.60-0.63 (m, 2H, H-2a cPr, H-3a cPr), 0.70-0.75 (m, 2H, H-2b cPr, H-3b cPr), 3.09 (bs, 1H, H-1 cPr), 3.84 (bs, 2H, H-5'), 4.03-4.05 (m, 1H, H-4'), 4.21-4.23 (m, 1H, H-3'), 4.64 (t, *J* = 5.9, 1H, H-2'), 5.93 (d, *J*_1_'_,2'_ = 5.9, 1H, H-1'), 7.86 (bs, 1H, NH), 8.24 (bs, 1H, H-2), 8.44 (s, 1H, H-8); ^13^C NMR (DMSO-*d*_6_) δ 6.9 (2C, C-2 cPr, C-3 cPr), 24.8 (C-1 cPr), 64.8 (d ^2^,*J*_CP_ = 5.0, C-5'), 71.5 (C-3'), 74.5 (C-2'), 84.6 (d ^3^,*J*_CP_ = 7.2, C-4'), 87.2 (C-1'), 120.2 (C-5), 139.6 (C-8), 149.9 (C-4), 153.0 (C-2), 155.3 (C-6); ^31^P NMR (DMSO-*d*_6_) δ -1.14; HRMS (ESI-TOF) *m/z* calcd for [C_13_H_18_N_5_O_7_P+H]^+^ 388.1022, found 388.1026.

#### *N* ^6^-Phenyladenosine 5'-monophosphate (11)

White powder (10 mg); *R*_t_ = 8.85 min (10-50% of CH_3_CN in 10 mM TEAA buffer); ^1^H NMR (DMSO-*d*_6_) δ 3.86-3.92 (m, 2H, H-5'), 4.09 (q, *J* = 3.3, 1H, H-4'), 4.26 (q, *J* = 3.2, 1H, H-3'), 4.70 (t, *J* = 5.5, 1H, H-2'), 6.00 (d, *J*_1_'_,2'_ = 5.5, 1H, H-1'), 7.02-7.06 (m, 1H, H-4 Arom.), 7.31-7.35 (m, 2H, H-3 Arom., H-5 Arom.), 7.93-7.96 (m, 2H, H-2 Arom., H-6 Arom.), 8.41 (s, 1H, H-2), 8.64 (s, 1H, H-8), 9.86 (bs, 1H, NH); ^13^C NMR (DMSO-*d*_*6*_) δ 64.8 (d ^2^,*J*_CP_ = 4.8, C-5'), 71.5 (C-3'), 74.5 (C-2'), 84.7 (d ^2^,*J*_CP_ = 7.1, C-4'), 87.4 (C-1'), 120.3 (C-5), 121.2 (2C, C-2 Arom., C-6 Arom.), 123.0 (C-4 Arom.), 128.8 (2C, C-3 Arom., C-5 Arom.), 140.1 (C-1 Arom.), 140.8 (C-8), 150.4 (C-4), 152.5 (C-2), 153.9 (C-6); ^31^P NMR (DMSO-*d*_6_) δ 1.20; HRMS (ESI-TOF) *m/z* calcd for [C_16_H_18_N_5_O_7_P+H]^+^ 424.1022, found 424.1006.

#### *N* ^6^-Phenylethyladenosine 5'-monophosphate (12)

White powder (38 mg); *R*_t_ = 10.38 min (10-50% of CH_3_CN in 10 mM TEAA buffer); ^1^H NMR (DMSO-*d*_6_) δ 2.95 (bs, 2H, CH_2_-Ph), 3.72 (bs, 2H, CH_*2*_-NH), 3.84-3.89 (m, 2H, H-5'), 4.03-4.07 (m, 1H, H-4'), 4.20-4.22 (m, 1H, H-3'), 4.65 (t, *J* = 5.4, 1H, H-2'), 5.93 (d, *J*_1_'_,2'_ = 5.4, 1H, H-1'), 7.16-7.21 (m, 1H, H-4 Arom.), 7.25-7.31 (m, 4H, H-Arom.), 7.78 (bs, 1H, NH), 8.24 (s, 1H, H-2), 8.44 (s, 1H, H-8); ^13^C NMR (DMSO-*d*_6_) δ 35.6 (CH_2_-Ph), 41.8 (CH_2_-NH), 64.8 (d ^2^,*J*_CP_ = 4.4, C-5'), 71.5 (C-3'), 74.4 (C-2'), 84.6 (d ^3^,*J*_CP_ = 7.6, C-4'), 87.3 (C-1'), 119.8 (C-5), 126.5 (C-4 Arom.), 128.7 (2C, C-Arom.), 129.1 (2C, C-Arom.), 139.7 (C-8), 140.0 (C-1 Arom.), 149.6 (C-4), 153.1 (C-2), 155.7 (C-6); ^31^P NMR (DMSO-*d*_6_) δ 1.08; HRMS (ESI-TOF) *m/z* calcd for [C_18_H_22_N_5_O_7_P+H]^+^ 452.1335, found 452.1329.

#### *N* ^6^-(2-carboxyethyl)adenosine 5'-monophosphate (13)

White powder (30 mg); *R*_t_ = 10.70 min (0-10% of CH_3_CN in 10 mM aq TEAA buffer); ^1^H NMR (MeOD-*d*_*4*_) δ 2.63 (t, *J* = 6.6, 2H, CH_*2*_-COO), 3.90 (bs, 2H, NH-CH_*2*_), 4.00-4.20 (m, 2H, H-5'), 4.27-4.29 (m, 1H, H-4'), 4.46 (dd, *J*_3_'_,4'_ = 3.1, *J*_2_'_,3'_ = 5.1, 1H, H-3'), 4.70 (t, *J* = 5.5, 1H, H-2'), 6.14 (d, *J*_1_'_,2'_ = 5.9, 1H, H-1'), 8.28 (s, 1H, H-2), 8.51 (s, 1H, H-8); ^13^C NMR (MeOD-*d*_*4*_) δ 37.6 (*C*H_2_-COO), 39.6 (NH-CH_2_), 65.8 (d ^2^,*J*_CP_ = 5.1, C-5'), 72.3 (C-3'), 76.4 (C-2'), 85.9 (d ^3^,*J*_CP_ = 8.9, C-4'), 88.9 (C-1'), 120.5 (C-5), 140.5 (C-8), 149.5 (C-4), 153.9 (C-2), 156.0 (C-6), 179.1 (COO); ^31^P NMR (MeOD-*d*_*4*_) δ 2.36; HRMS (ESI-TOF) *m/z* calcd for [C_13_H_18_N_5_O_9_P+H]^+^ 420.0920, found 420.0918.

#### *N* ^6^-(3-carboxypropyl)adenosine 5'-monophosphate (14)

White powder (32 mg); *R*_t_ = 8.98 min (0-20% of CH_3_CN in 10 mM TEAA buffer); ^1^H NMR (D_2_O) δ 1.80-2.11 (m, 2H, CH_*2*_-CH_2_-COO), 2.38 (t, *J* = 7.6, 2H, CH_*2*_-COO), 3.63 (bs, 2H, NH-CH_*2*_), 4.10-4.23 (m, 2H, H-5'), 4.37-4.47 (m, 1H, H-4'), 4.55 (dd, *J*_3_'_,4'_ = 3.7, *J*_2_'_,3'_ = 5.2, 1H, H-3'), 4.81 (t, *J* = 5.4, 1H, H-2'), 6.17 (d, *J*_1_'_,2'_ = 5.7, 1H, H-1'), 8.27 (s, 1H, H-2), 8.48 (s, 1H, H-8); ^13^C NMR (D_2_O) δ 28.1 (*C*H_2_-CH_2_-COO), 37.2 (*C*H_2_-COO), 43.2 (NH-CH_2_), 66.9 (d ^2^,*J*_CP_ = 4.9, C-5'), 73.1 (C-3'), 77.0 (C-2'), 86.6 (d ^3^,*J*_CP_ = 8.7, C-4'), 89.6 (C-1'), 121.5 (C-5), 141.8 (C-8), 150.6 (C-4), 155.3 (C-2), 157.1 (C-6), 185.0 (COO); ^31^P NMR (D_2_O) δ 2.18; HRMS (ESI-TOF) *m/z* calcd for [C_14_H_20_N_5_O_9_P+H]^+^ 434.1077, found 434.1062.

### Cloning, Overexpression and Purification of the N-terminal His-tagged DNPH1s

The human *dnph1* gene was amplified from the mammalian gene ATCC® Number: MGC-19540 using oligonucleotides olhumrclnde: 5’-ATGAATTCCATATGGCTGCTGCCATGGTGCCG-3’ and olhumrclHind 5’- CCCAAGCTTTCAAGTGGTTGGGTCAGGGGAG -3’ in a standard PCR reaction. The PCR product was digested with NdeI and HindIII, purified and ligated to pET28a digested with the same restriction enzymes. After transformation into *E.coli* DH5α strain, the plasmid DNA of several colonies was extracted and purified and sequenced. The pET28a rat *dnph1* [24] and pET28a human *dnph1* plasmids were used to transform *E.coli* Bli5 strain. Culture conditions and induction were performed as described by Konto-Ghiorghi et al [24]. Frozen cells resuspended in 40 mL of extraction/wash buffer (50 mM Na_2_HPO_4_, NaH_2_PO_4_, 300 mM NaCl pH 7.0) were lysed using a French press at 14000 p.s.i. The lysate was centrifuged at 25000 g for 30 min at 4°C. The supernatant was loaded on a 6 mL TALON (BD Bioscience) resin column previously equilibrated with the same buffer. After washing, DNPH1 was eluted with 150 mM imidazole. Fractions containing DNPH1 were pooled and dialyzed against so50 mM Na_2_HPO_4_, NaH_2_PO_4_, pH 6.0). The purity was checked by SDS PAGE electrophoresis and by measuring the specific activity. Purified His-tagged DNPH1s in 50 mM sodium phosphate buffer, pH 6.0, were stored at -20°C.

### Kinetic measurements

The enzymatic activity of the rat DNPH1 (5 µM) was determined spectrophotometrically by incubating the enzyme with dGMP (100 µM) and by following the production of 2-deoxyribose 5-phosphate as described previously [24]. The activity of human DNPH1 was determined by incubating the enzyme (14 µM) with dGMP (200 µM) and by following the production of guanine (G) by RR-HPLC on a C18 reverse phase column (ZORBAX Eclipse XDB-C18, 2.1x50 mm, 1.8 µm) using a flow rate of 0.25 mL/min and a 1-12% linear gradient of acetonitrile in 20 mM TEAA buffer at pH 7 over 3.50 min. The retention time of G is 1 min and that of dGMP is 2.8 min. The initial velocity of the reaction was measured either at a variable concentration of dGMP, both in the absence and presence of inhibitors, or at a fixed concentration of dGMP and variable concentrations of inhibitors, allowing investigation of the nature of the inhibition, i.e. competitive in all cases.

### Isothermal titration calorimetry

ITC was performed in a MicroCal VP-ITC calorimeter at 25°C. Protein samples were prepared as indicated above except that they were further purified by gel filtration on a Superdex 200 column. Following thermal equilibration, titrant additions were made at 600-s intervals to the 1.41 mL protein samples by adding 5 µL aliquots of 300 µM compounds to protein samples ([DNPH1] = 15 µM) in 25 mM Na_2_HPO_4_, NaH_2_PO_4_, pH 6.0, 25 mM NaCl and 2 mM Tris[2-carboxyethyl] phosphine (TCEP). In the case of compound **14** (*K*_i_ = 11.2 µM), a concentration of 1 mM was used and [DNPH1] = 20 µM. The heat of dilution obtained from injecting the ligand into buffer was substracted before fitting. Heat effects were integrated with ITC Origin 7.0 software and a best fit was found using a single binding-site model.

### Crystallization and structure refinement

Crystal growth conditions have been previously described [18] and are briefly reported here. They were refined from a primary sparse screen, Anions, pHClear and (NH_4_)_2_SO_4_ from Qiagen. Starting from selected conditions, having the best crystal-diffraction quality, further refinement was done with 1.1 to 1.3 M (NH_4_)_2_SO_4_ and 1.2 to 1.4 M Li_2_SO_4_, in mild basic buffer (100 mM Tris pH 7.4 to 8.0) at the protein concentration of 12 mg/mL in 50 mM citrate buffer pH 7.5, which gave rise to diffracting crystals from 2.0 and up to 1.6 Å resolution of rat DNPH1 in complex with the compounds **1**, **5**, **7** and **12**. The other compounds, **8**, **9**, **10**, **11** and **13** failed to co-crystallize and our attempts to soak those ligands failed to displace the original one. The crystal structures were solved by molecular replacement using Molrep [30] and as a template the recently solved dimeric structure of rat DNPH1 (PDB4FYI; [18]). The first refinement steps of the structures of Rat DNPH1 in complex with **1**, **5** and **7** were performed using Refmac5 [31] with iterative model building of the protein alone using Coot [32]. The ligand chemical structures were designed using the PRODRG server [33] and were placed in the density. Water molecules were added automatically using Coot and the structures of the complexes were further refined using Refmac5. Space groups and refinement statistics are given in Table 1.

**Table 1:** Data collection, phasing, and refinement statistics for rat DNPH1 structures.

	DNPH1-**7**	DNPH1-**1**	DNPH1-**5**
Beamline	ID14-4	FIP-BM30	ID23-2
No. of crystals	1	1	1
Space group	*P*12_1_1	*P*12_1_1	*P*12_1_1
Unit-cell parameters			
a (Å)	32.7	32.2	32.2
b (Å)	100.6	96.6	95.5
c (Å)	79.7	79.0	79.3
ß (°)	101.8	101.6	101.6
No. molecules in asymmetric unit	4	4	4
Wavelength (Å)	0.9795	0.9798	0.8726
Resolution (Å)	1.69 (1.79-1.69)	2.24 (2.39-2.24)	1.90 (2.00-1.90)
Rmerge (%)#	6.5 (11.5)	5.0 (10.7)	8.6 (35.6)
<I/σI>	5.4 (2.6)	11.1 (6.7)	7.9 (2.1)
Completeness (%)†	93.8 (75.8)	92.0 (65.4)	98.6 (96.6)
Multiplicity †	3.9 (2.8)	3.4 (2.8)	2.2 (2.1)
Wilson *B* factor (Å^2^)	14.9	17.2	14.6
**Refinement**			
Resolution (Å)	27.02-1.69	38.71-2.24	23.86-1.90
No. of reflections	50316	19911	33689
Rwork/Rfree (%)‡	17.6/21.2	17.4/22.7	23.2/28.6
No. Atoms			
Protein	4377	4274	4176
Ligand	112	116	112
Ions	10	-	-
Water	497	183	243
*B* factors (Å^2^)			
Protein	15.3	15.6	16.9
Ligand	15.3	17.0	15.0
Ions	53.7	-	-
Water	28.1	19.0	24.1
R.m.s deviations §			
Bond lengths (Å)	0.009	0.008	0.009
Bond angles (°)	1.41	1.15	1.90
**Ramachandran (%)**			
most favoured	92.7	93.5	92.8
additional allowed	7.3	6.5	7.2
disallowed regions	0	0	0

# ∑hkl∑i|Ihkl,i – Iaverage,hkl|/|∑hkl∑i|Ihkl,i|x100.

† Values in parenthesis stands for the outer shell of resolution range.

‡ Rfree is calculated on a subset of reflections that are not used in the refinement (5 %).

§ Deviation from ideal values.

## Results

### Catalytic activity of human DNPH1

Since the majority of data concerning DNPH1 were performed with human cells, tissues and tumors, we decided to purify and characterize the human DNPH1.

Initial velocity experiments were carried out at variable concentrations of the six 2'-deoxynucleoside 5’-monophosphates, dAMP, dCMP, dGMP, dIMP, dTMP and dUMP (Table 2). The kinetic parameters indicate that human DNPH1 has a *K*_*m*_ of 57 µM for dGMP, and a *V*_*max*_ of 0.005 units.mg^-1^ corresponding to a *k*_*cat*_ of 0.002 s^-1^. As reported previously for the rat DNPH1 enzyme [24], purine deoxynucleotides have a higher affinity for the human DNPH1 than pyrimidine deoxynucleotides. The *K*_*m*_ of human DNPH1 for dCMP (2.5 mM), dUMP (7.8 mM) and dTMP (23 mM) are high compared to dGMP (57 µM), dAMP (97 µM) and dIMP (104 µM). Whereas rat and human DNPH1s have different *k*_*cat*_, they have similar *K*_*m*_ values. These differences of *k*_*cat*_ should be interpreted with caution since both enzymes were produced in *Escherichia coli*. However, we cannot exclude that post-translational modifications enhance or regulate the enzymatic activity in the cellular context. 

**Table 2:** Kinetic parameters of the human DNPH1 with different 2'-deoxynucleosides 5’-monophosphates as substrates.

	***k*_*cat*_ (10^-3^ s^-1^)**	***K*_*m*_ (mM)**	***k*_*cat*_ /*K*_m_ (M^-1^. s^-1^)**
**dGMP**	2.0 ± 0. 2	57 ± 12	35 ± 2
**dAMP**	2.5 ± 0.8	97 ± 14	25 ± 2
**dIMP**	1.6 ± 0.1	104 ± 19	15 ± 3
**dCMP**	8.3 ± 0.4	2500 ± 207	3.3 ± 0.3
**dUMP**	200 ± 20	7800 ± 1760	25 ± 6
**dTMP**	25 ± 2	23000 ± 3050	1.0 ± 0.2

*K*_m_ were obtained from double reciprocal plots of initial velocity measurements. For each dNMPs five different concentrations were used.

**Figure 1:**
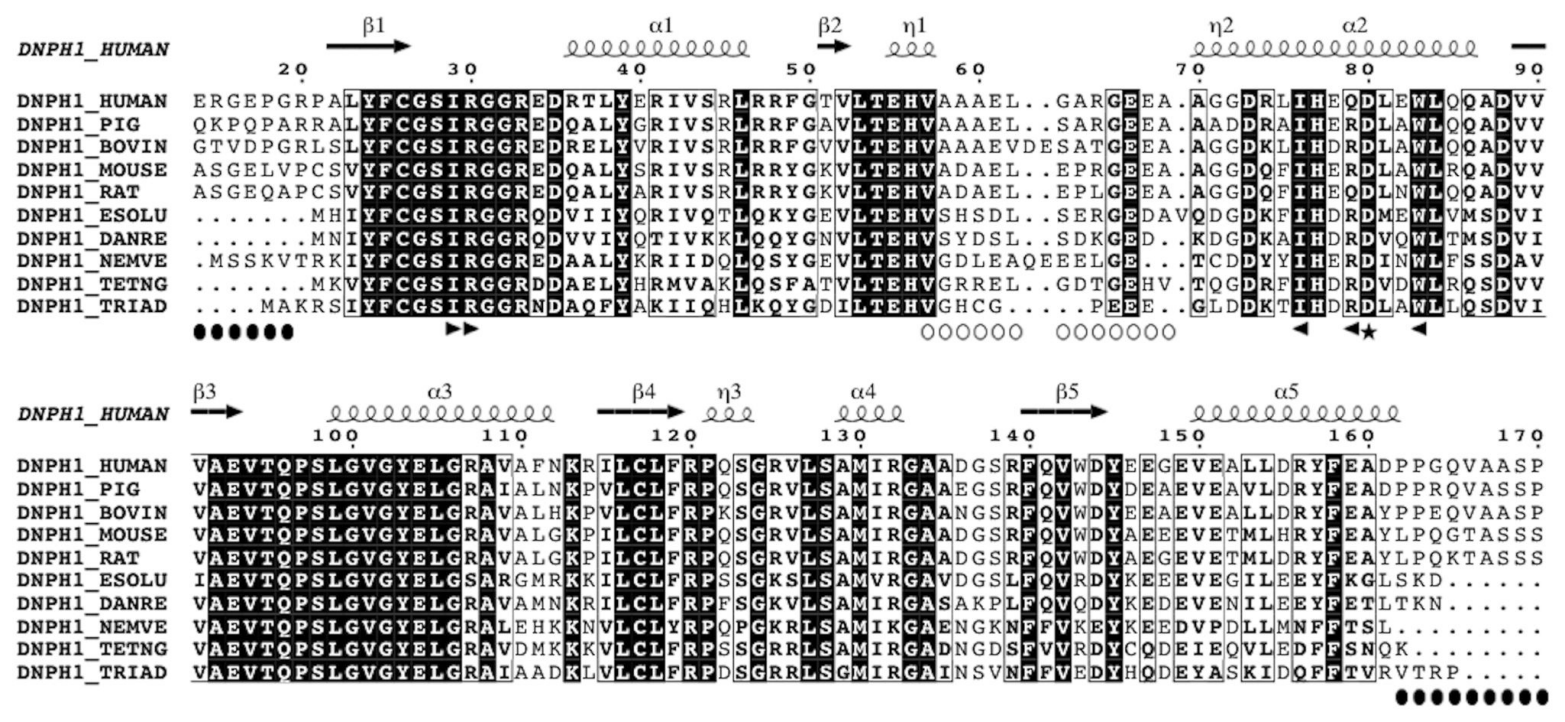
Sequence alignment of animal DNPH1s. Sequences of DNPH1 enzymes from various species (ranging from human to fish) have been refined using ViTO (39) to take into account the known crystal structures of the rat DNPH1. Secondary structures are shown above the sequence alignment. Sequence numbering corresponds to the rat sequence. Black circles underline the regions deleted in the crystallized recombinant enzyme. Open circles underline the long flexible loop surrounding the nucleotide binding site. Black arrows indicate the position of the residues (I18’, R19’, F64’, I65’, Q68) whose side chains interact with the *N* ^6^-substituents in the solved crystal structures. The figure was generated by ESPRIPT (40).

These data showed, however, that both enzymes possess rather similar functional features in agreement with their highly similar sequences (Figure 1). This observation validates the use of rat DNPH1 to study the protein-ligand interactions by X-ray crystallography (see below).

### DNPH1 inhibition by cytokinin ribosides 5’-monophosphates (1-6) and related **N** ^6^-substituted AMP derivatives (7-14)

We have previously shown that purine ribonucleotides inhibit the rat DNPH1 enzyme and that small substituents on the purine ring are well tolerated [25]. Structure elucidation of the rat DNPH1 prompted us to investigate the impact of larger substituents in position N6 of AMP on the binding affinity for mammalian DNPH1 enzymes.

**Figure 2:**
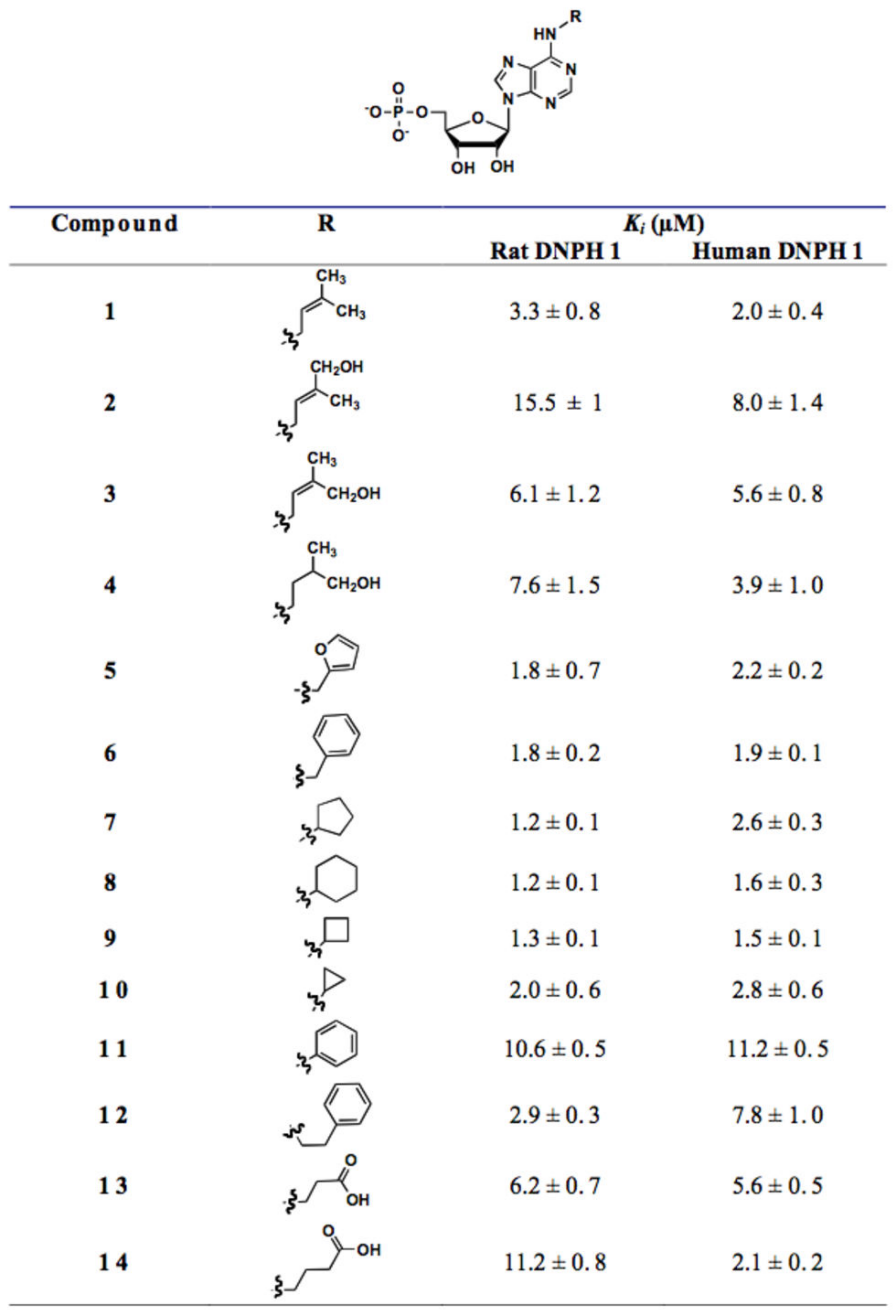
DNPH1 inhibition by cytokinin ribosides 5’-monophosphates and *N* ^6^-substituted AMP.

Different cycloalkyls, saturated and unsaturated alkyl chains with aromatic or functional groups were considered at the N6 position. As summarized in Figure 2, all tested compounds are better inhibitors than AMP (*K*_*i*_ = 40 µM) [25], with inhibition potencies in the micromolar range against rat DNPH1 (*K*_*i*_ ranging from 1.2 to 15.5 µM depending on the substitution).

The size of the cycloalkyl group (from 3- to 6- membered ring) appears to have little influence on the inhibition potency, as well as the nature of aromatic rings (methylfuryl **5**, benzyl **6**, and phenylethyl **12**), as confirmed by the similar *K*_*i*_ values (from 1.8 to 2.3 µM). Surprisingly, AMP bearing a phenyl ring (compound **11**) revealed a moderate *K*_*i*_ (10.6 µM) illustrating the benefit of a methylene group between N6 and the cycloalkyl or aromatic group.

All these substitutions significantly improved the inhibition potency towards rat DNPH1 as compared to AMP (up to 40 fold higher). This could be explained by a contribution of the hydrophobic substituents. 

By contrast, the presence of polar functional groups (such as alcohol or carboxylic acid) significantly decreased the inhibition of DNPH1s. Indeed, whereas compound **1** is a good inhibitor (*K*_*i*_ of 3.3 and 2.0 µM for rat and human DNPH1, respectively), the presence of a hydroxyl group on the C4 chain (zeatin derivatives **2**, **3** and **4**) resulted in a significant decrease in activity (up to 5-fold for **2** compared to **1**).

These results were quite surprising regarding the largely solvent-accessible active site where the nucleobase is sandwiched between two hydrophobic isoleucine residues (I18 and I65) while its N1/N6 edge faces the solvent [18]. 

### Binding affinity of the **N** ^6^-substituted AMP derivatives

**Figure 3:**
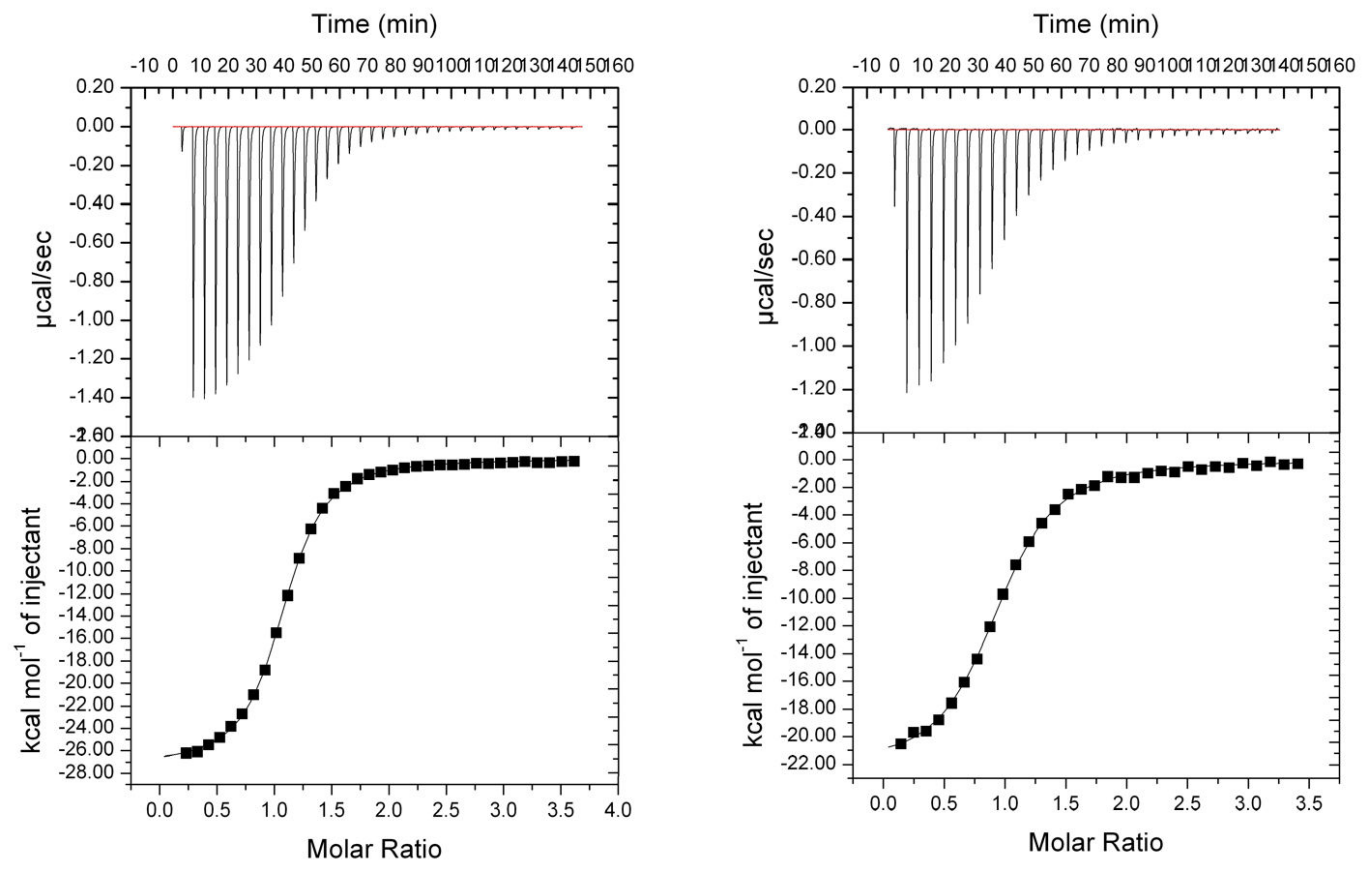
Representative isothermal titration of rat (left) and human (right) DNPH1with inhibitor 6, best-fitted with one-site binding model.

To further characterize the ligand-DNPH1 interactions, the thermodynamic binding parameters of some *N* ^6^-substituted AMP derivatives to rat and human DNPH1s were determined using isothermal titration calorimetry (ITC) (Table 3). Representative ITC results and fitting curves for inhibitor **6** binding to rat and human DNPH1 are shown in Figure 3. All *N* ^6^-substituted AMP tightly bind rat DNPH1 (*K*_d_ values ranging from 0.4 to 2.6 µM), with the exception of **14** (*K*_d_ of 9.4 µM). Thus, the free energies are lower as compared to GMP (-6.8 kcal mol^-1^) [25] suggesting additional interactions with the protein. Most of the substituents at position 6 improved the enthalpic term compared to GMP [25]. The beneficial effect is more pronounced for the aromatic substituents (compounds **5** and **6**). By contrast, no gain in entropy was observed. Only the cyclopentyl group (compound **7**) showed a favorable entropic contribution, although the ∆S term remains negative. 

**Table 3:** Dissociation constants *K*_d_ of human and rat DNPH1 with some cytokinin ribosides 5’-monophosphates and *N* ^6^-substituted AMP as ligands at T = 298 K.

**Rat DNPH1**	***K*_d_**(µM)	**∆H(kcal.mol^-1^)**	**∆S(cal.K^1^.mol^-1^)**	**-T∆S (kcal.mol^-1^**)	**∆G (kcal.mol^-1^)**
**1**	2.6 ± 0.1	-15.9 ± 0.1	-27.9	8.3	-7.6
**5**	0.8 ± 0.1	-25.7 ± 0.1	-58.3	17.4	-8.3
**6**	0.4 ± 0.1	-27.4 ± 0.1	-62.4	18.6	-8.8
**7**	0.6 ± 0.1	-10.7 ± 0.1	-7.5	2.3	-8.5
**8**	0.5 ± 0.1	-19.9 ± 0.1	-37.6	11.2	-8.7
**14**	9.4 ± 0.1	-12.4 ± 0.1	-18.5	5.5	-6.9
**human DNPH1**	***K*_d_** (µM)	**∆H (kcal.mol^-1^)**	**∆S (cal.K^-1^.mol^-1^)**	**-T∆S (kcal.mol^-1^**)	**∆G (kcal.mol^-1^)**
**1**	2.1 ± 0.2	-10.2 ± 0.1	-8.2	2.4	-7.8
**5**	1.4 ± 0.1	-20.1 ± 0.1	-42.4	12.6	-7.5
**6**	0.7 ± 0.1	-26.1 ± 0.1	-59.5	17.7	-8.4
**7**	1.4 ± 0.4	-6.1 ± 0.1	6.2	-1.8	-8.0
**14**	5.5 ± 0.1	-9.8 ± 0.1	-8.8	2.6	-7.2

Data were collected in 25 mM Na2HPO4, NaH2PO4, pH 6.0, 25 mM NaCl, 2mM Tris[2-carboxyethyl] phosphine (TCEP) and [DNPH1] = 15 µM, [compounds] = 300 µM except for [compound **14**] = 1 mM and [DNPH1] = 20 µM. Data were corrected for ligand heats of dilution and deconvoluted using the Microcal Origin software. The results of the least-squares fit assuming a single site binding model are displayed. Experiments were done in duplicate.

These binding parameters are very similar to those obtained with human DNPH1 (Table 3). 

**Figure 4:**
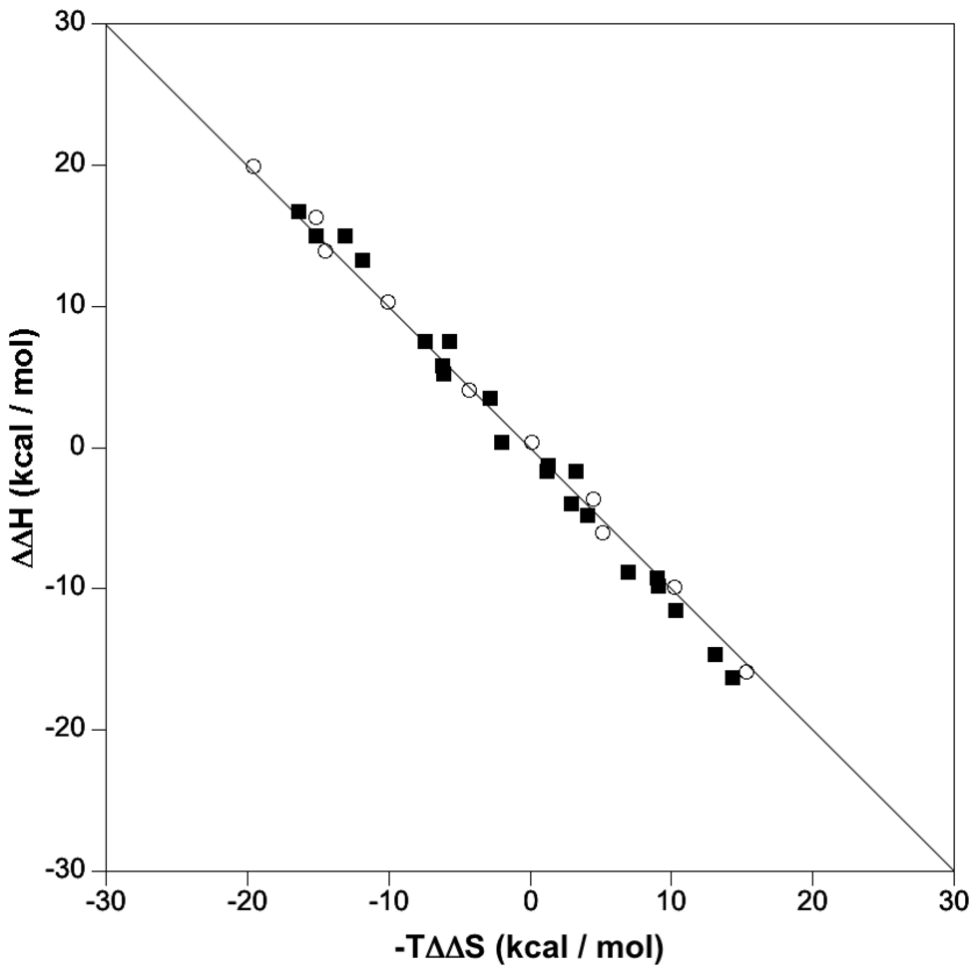
∆∆H versus -T∆∆S plot of experimental differences, compiled from the ITC data of Table 3 at 298 K, for pairs of ligands binding to human DNPH1 (open circles) and rat DNPH1 (black squares). The line corresponds to full enthalpy-entropy compensation (∆∆H = T∆∆S).

In order to diminish the experimental constraints on ITC measurements, the thermodynamic data were transformed into ∆∆-plots according to Olsson [34]. A strict compensation is observed since T∆∆S ≈ ∆∆H and consequently ∆∆G ≈ 0 (Figure 4). Thus, there is no gain of ∆G for any of the ligand and no significant change in the order of magnitude of *K*_d_. 

In an attempt to rationalize these biochemical and thermodynamic data, a structural study was performed.

### Crystal structure of rat DNPH1 in complex with **N** ^6^-substituted AMP derivatives

In a previous study, we solved the crystal structure of rat DNPH1 bound to three distinct nucleotide analogs [18]. These crystal structures revealed the tight recognition of the phosphate group as well as the ribose moiety of the nucleotide by DNPH1. Indeed, an intricate network of several hydrogen bonds formed between the phosphate group and the protein that involves the side chains of two serines (Ser87 and Ser17’) and the backbone N atoms of Ile18, Arg19, Gly20 and Gly89. The ribose tightly interacts with Gly16, Gly89, Glu93 and Met119’. The two hydroxyls O2’ and O3’ atoms of the ribose are hydrogen-bonded to the carboxylate group of Glu93. This indicates that there is apparently little room for chemical variations in this part of the ligand to improve the affinity for this target. In parallel, these complexes have revealed that the N6 position was amenable to significant substitutions. However, as discussed above, the rationale to further guide the design of enhanced inhibitors was not obvious. The crystal structure of some cytokinins (or close analogs) bound to rat DNPH1 was expected to provide brighter insights.

High-resolution data were obtained for three complexes while the other ligands failed to provide us with decent crystals. The new structures were all obtained in the same monoclinic space group (*P*12_1_1) that differs from the orthorhombic form previously described despite the similar conditions used to grow the crystals. These results suggest that we can survey the impact of the different crystal packing on the protein conformation. Having one complex - rat DNPH1-**7** - in common in the two available space groups, we could conclude that the absence of electron density for the long loop (residues 47-59) was due to its intrinsic flexibility rather than packing issue. This is in agreement with published NMR data [27]. This is reinforced by the observed flexibility of this loop in the four independent monomers in the asymmetric unit of both space groups. This particular feature may explain the small influence of the *N* ^6^-substituent on the inhibitory activity of the various cytokinins against DNPH1.

**Figure 5:**
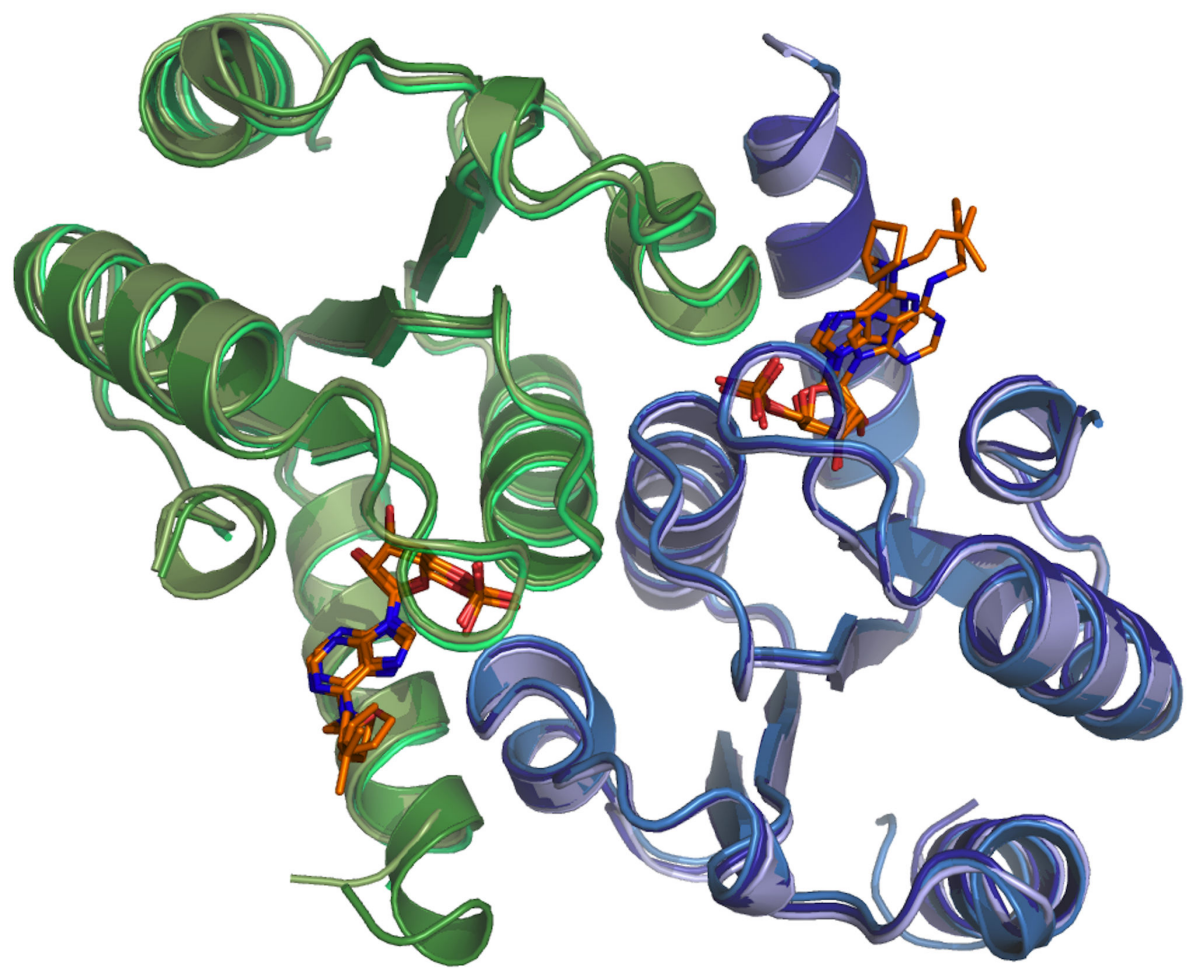
Superposed crystal structure of rat DNPH1 in complex with nucleotides *N* ^6^-cyclopentyl AMP, *N* ^6^-furfuryl AMP and *N* ^6^-isopentenyl AMP (compounds numbered 7, 5 and 1 in the text, respectively). One dimer of each complex is shown in blue/green ribbons. The corresponding ligands are shown as orange sticks. The figures were generated by Pymol (www.pymol.org).

**Figure 6:**
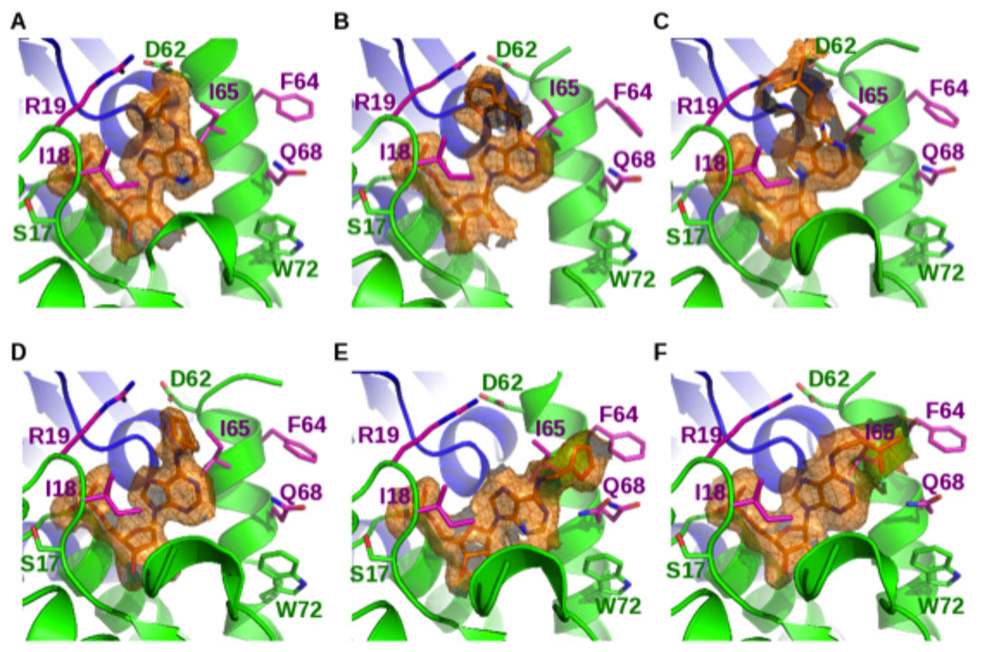
The active site of the DNPH1 structures in complex with nucleotides *N* ^6^-cyclopentyl AMP (A, D), *N* ^6^-furfuryl AMP (B, E) and *N* ^6^-isopentenyl AMP (C, F). The ligand conformers correspond to two independent monomers in the crystal structures of rat DNPH1 in the monoclinic forms (this study; panels A, B, C, E, and F) and the previously published orthorhombic form (PDB4FYI) in the first panel D). Omit maps were computed by omitting the ligand at the last refinement step before computing the electron density. The density of the bound ligands were contoured at the 1.0 s level and shown as orange surfaces and meshes. Backbones are represented as ribbons (front monomer in green and back monomer in blue) and ligands as sticks. Important side chains were drawn as sticks. Side chains contacting the *N* ^6^-substituents (I18, R19, F64, I65 and Q68) are shown in pink and some other residues that make up the active site (S17, D62, W72) are in green. The figures were generated by Pymol (www.pymol.org).

Indeed, the three novel crystal structures showed little structural variation for protein structure. The two dimers in the asymmetric unit from these three crystal structures can be superimposed (as exemplified in Figure 5) with an averaged r.m.s.d. of 0.5 Å, only slightly above the experimental error (~0.3 Å). In all these structures, the phosphate groups and the ribose moieties showed the same tight interactions with the protein side chains and backbone atoms as previously described [18]. Similarly, the adenine moiety showed a conserved orientation constrained by the two sandwiching isoleucines I18 and I65 in the three complexes. Conversely, the three distinct *N* ^6^-derivatizations appeared to be mobile in the crystal structures and to occupy distinct locations depending on the monomer considered. Two main families of orientation are detected whatever the chemical derivatization (isopentenyl, furfuryl and cyclopentyl). In some monomers, the *N* ^6^-substitution points toward a hydrophobic subpocket formed by the side chain of isoleucine I18 and part of the side chain of arginine R19 (Figure 6a, b and c) while in other monomers, the substitution points rather outward and interacts mainly with isoleucine I65 and rather weakly with the glutamine Q68 in the case of compound **5** (Figure 6d, 6e and 6f). Accordingly, whatever the *N* ^6^-substitution, rather weak van der Waals contacts are observed and little stabilization of the complexes occurred. As the three distinct complexes are likely to represent good templates for the modeling of the binding mode of the other ligands studied here (with **7** for cycloalkyl, **5** for aromatic cycles and **1** for alkyl substituents), one may conclude that alternating conformations can be predicted for all the *N* ^6^-substituted AMP bound to mammalian DNPH1. None of the tested substition can fit a buried sub-pocket to maximize additional interactions. This suggests that the few favorable interactions observed in one configuration or another (inward for 7 or 1 and outward for **5**) are not sufficient to dramatically alter the binding affinity. This is in agreement with our thermodynamic data (compiled in Table 3) showing enthalpy-entropy compensation. Any additional but weak interactions made at the surface of the protein by a given *N* ^6^-substituent are counterbalanced by the entropy cost of its stabilization (or desolvatation in some cases such as compound **5**) in one orientation.

## Discussion

The three related phytohormones, *N* ^6^-isopentenyladenosine, *N* ^6^-benzyladenosine and *N* ^6^-furfuryladenosine display anti-cancer activity both *in vitro* and *in vivo* and induce apoptosis through caspase-3 activation [4,5]. These nucleoside derivatives do not act by interfering with DNA synthesis as most other nucleoside analogs; rather, they accumulate intracellularly as mononucleotides (the di- and tri-phosphate being traces) [14,35]. This particular mode of action resembles that described for triciribine [15,16].

Nevertheless, the actual molecular targets are still unknown and the precise mechanism of action of these cytokinin ribosides remains to be determined. By similarity with triciribine, Akt might be one possible target. Indeed, *N* ^6^-isopentenyladenosine suppresses the nuclear factor kappaB (NF-κB) pathway and inhibits the Akt activation [36]. 

The kinetic, thermodynamic and structural data reported here indicate that DNPH1 may be a potential cytokinin target. Some cancer cell lines are killed by cytokinins at low micromolar concentrations [37,38], a concentration range that inhibits the activity of DNPH1. The differences of values between the lethal concentrations in the different cell lines and the *K*_*i*_ values for DNPH1 may be explained by the phosphorylation capacity of ADK [14], the existence of an adenosine deaminase-like enzyme ADAL1 that catalyzes the removal of different alkyl groups at position 6 from *N* ^6^-substituted purine and 2-aminopurine nucleosides 5’-monophosphates [39] and the level of *dnph1* expression.

It is likely that cytokinins may also bind other targets such as the lactate dehydrogenase LDH-A, which is the form of LDH expressed in many cancer cells [40]. It is interesting to note that inhibition of LDH-A slows the growth of xenografts tumours in mice and induces tumour regression [41]. Whereas the overexpression of DNPH1 nor LDH-A alone do not induce tumorigenesis, cells expressing both DNPH1 and LDH-A form tumors after injection into nude mice [42]. It is therefore tempting to hypothesize that *N* ^6^-substituted AMP derivatives might have to inhibit both DNPH1 and LDH-A to efficiently inhibit cell growth. 

Another argument in favor of other targets than DNPH1 for the inhibition of cell growth is, for example, provided by the identification of human tyrosyl-DNA phosphodiesterase 1 (TDP1), SMAD3 or PAX8 with micromolar affinity for *ortho-*topolin riboside, the most active cytokinin reported so far, (See Pubchem CID 13506406 Compound BioActivity Data). 

These data suggest that an intricate network of activation and inhibition may play a role in the anti-cancer activity of triciribine or other nucleoside analogs such as cytokinins.

Stringent affinity chromatography or drug affinity responsive target stability (DARTS) may help to identify cytokinin protein targets [43]. 

Our current study shows that cytokinin ribosides 5’-monophosphates inhibit mammalian DNPH1. Thermodynamic and structural characterization of DNPH1 in complex with *N* ^6^-substituted AMP suggests further modifications. Derivatizations at the N6 position are currently underway and are expected to compensate the observed alternation in binding modes. These chemical tools will contribute to elucidate the role of DNPH1 in cellular proliferation.
