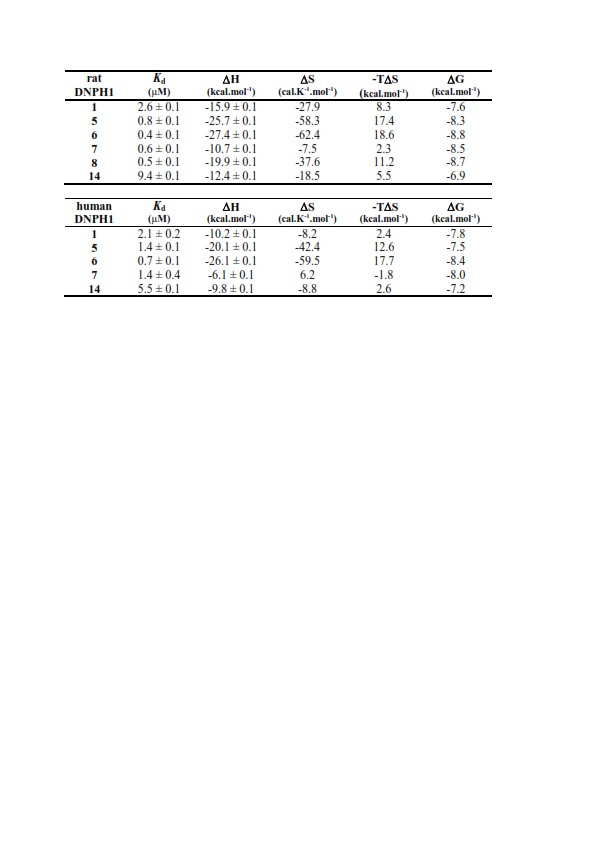# Correction: *N* ^6^-Substituted AMPs Inhibit Mammalian Deoxynucleotide *N*-Hydrolase DNPH1

**DOI:** 10.1371/annotation/c529909c-9d88-4c03-9262-ff5c61b96a36

**Published:** 2014-01-29

**Authors:** Claire Amiable, Sylvie Pochet, André Padilla, Gilles Labesse, Pierre Alexandre Kaminski

An error occurred in the fourth column of Table 2 in which the variables were switched. (M^-1^. s^-1^) should instead read (s^-1^.M^-1^). Please see the correct version of Table 2 here: 

**Figure:**
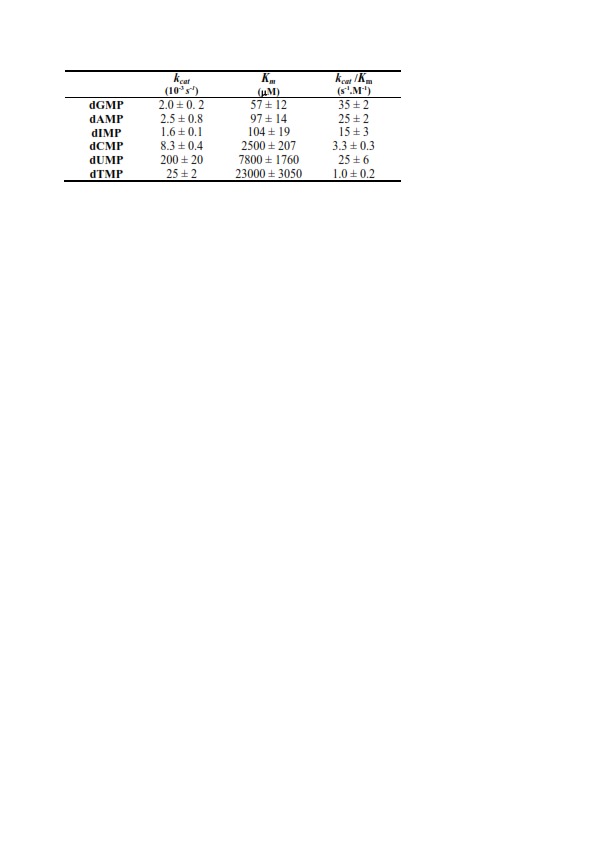


An error also occurred in the first row, fourth column of Table 3 in which a negative sign disappeared. ∆S(cal.K^1^.mol^-1^) should instead read ∆S(cal.K^-1^.mol^-1^). Please see the correct version of Table 3 here: 

**Figure:**